# Aberrant Functional Organization within and between Resting-State Networks in AD

**DOI:** 10.1371/journal.pone.0063727

**Published:** 2013-05-07

**Authors:** Jinyu Song, Wen Qin, Yong Liu, Yunyun Duan, Jieqiong Liu, Xiaoxi He, Kuncheng Li, Xinqing Zhang, Tianzi Jiang, Chunshui Yu

**Affiliations:** 1 Department of Radiology, Tianjin Medical University General Hospital, Tianjin, China; 2 National Laboratory of Pattern Recognition, Institute of Automation, The Chinese Academy of Sciences, Beijing, China; 3 Department of Neurology, Xuanwu Hospital of Capital Medical University, Beijing, China; 4 Department of Radiology, Xuanwu Hospital of Capital Medical University, Beijing, China; Banner Alzheimer’s Institute, United States of America

## Abstract

Altered functional characteristics have been reported in amnestic mild cognitive impairment (aMCI) and Alzheimer’s disease (AD); nonetheless, comprehensive analyses of the resting-state networks (RSNs) are rare. This study combined multiple imaging modalities to investigate the functional and structural changes within each RSN and between RSNs in aMCI/AD patients. Eight RSNs were identified from functional MRI data from 35 AD, 18 aMCI and 21 normal control subjects using independent component analysis. We compared functional connectivity (FC) within each RSN and found decreased FC in the several cognitive-related RSNs in AD, including the bilateral precuneus of the precuneus network, the posterior cingulate cortex and left precuneus of the posterior default mode network (DMN), and the left superior parietal lobule of the left frontoparietal network (LFP). We further compared the grey matter volumes and amplitudes of low-frequency fluctuations of these regions and found decreases in these measures in AD. Importantly, we found decreased inter-network connectivity between the visual network and the LFP and between the anterior and posterior DMNs in AD. All indices in aMCI patients were numerically between those of controls and AD patients. These results suggest that the brain networks supporting complex cognitive processes are specifically and progressively impaired over the course of AD, and the FC impairments are present not only within networks but also between networks.

## Introduction

Alzheimer’s disease (AD) is the most common type of dementia in the elderly population and is clinically characterized by an early impairment of memory function, followed by a slow progression of additional cognitive deficits that ultimately develop into overt dementia. AD is a genetically complex and irreversible neurodegenerative disease of the central nervous system with an insidious onset, but its pathogenesis is poorly understood, and effective therapies remain elusive. Mild cognitive impairment (MCI), especially amnestic MCI (aMCI), is considered an intermediary state between normal cognition and AD [1], [2]. Much evidence has shown that AD is associated with cortical atrophy and a disruption of metabolism and function [3], [4]. Neuropsychological studies have shown that patients with AD exhibit impairments in multiple cognitive domains, such as episodic memory, execution, attention, visuospatial orientation, and verbal ability [5], which suggests that AD is a disorder that causes deficits in multiple neural networks [6]. A better understanding of the neurobiology of AD requires investigations at the brain network level.

Independent component analysis (ICA) of resting-state functional MRI (fMRI) data is intrinsically a multivariate, data-driven method that extracts from the BOLD time series a number of independent resting-state networks (RSNs) (spatial components), each with its own specific time course [7], [8]. The common RSNs include the default mode network (DMN), the frontoparietal network (FPN), the central-executive network (CEN), the visual network (VN), the auditory network (AN), and the sensorimotor network (SMN) [9]–[14]. Among these RSNs, the DMN has been extensively investigated and found to be impaired in MCI/AD patients [15]–[21]. Recently, several studies have revealed that MCI or AD patients also show functional changes in other RSNs, such as the attention-related networks [22],[23], the frontal cognitive networks [24], [25], the self-referential network [26], and the motor and visual processing networks [27], [28]. Currently, we still have a limited understanding of the changes in the functional architecture of the non-DMN networks in AD/MCI patients. More importantly, whether the FCs between different RSNs are altered in MCI/AD patients remains largely unknown, although a recent region of interest (ROI)-based FC study revealed decreased network connectivity in AD [29], and a resting-state fMRI study discovered altered directional connectivity among RSNs in AD [30]. FC between different brain functional networks that can be considered a larger scale of FC is termed functional network connectivity (FNC) [31]. FNC can also be investigated with ICA because different ICA components are maximally spatially independent, but their corresponding time courses can show considerable amounts of temporal dependency. Thus, FNC analysis can be performed by analyzing the dependencies among ICA time courses.

In the present study, we performed a comprehensive analysis on both structural and functional MRI data from normal controls (NC) and patients with aMCI/AD to answer the following questions. Which RSNs are selectively impaired in AD, and are these RSNs also impaired in aMCI [25]? Do brain areas with impaired FC in a RSN in AD also exhibit alterations in grey matter volume (GMV) or regional brain activity as assessed by the amplitude of low-frequency fluctuations (ALFF)? Which functional changes are independent of the structural changes [32], [33]? And are FNCs also changed in MCI/AD patients [29], [30]?

## Materials and Methods

### Subjects

This study was approved by the Medical Research Ethics Committee of Xuanwu Hospital of Capital Medical University, and written informed consent was obtained from all participants. Eighty-seven older subjects underwent a standard dementia screening that included acquisition of a medical history, physical and neurological examinations, screening laboratory tests, extensive neuropsychological testing and brain MRI. Cognitive function was evaluated with the mini-mental state examination (MMSE), and the degree of dementia was determined by the clinical dementia rating scale (CDR). The diagnosis of AD met the NINCDS-ADRDA (National Institute of Neurological Communicative Disorders and Stroke and the Alzheimer’s Disease and Related Disorders Association) criteria for “probable AD” [34]. The diagnosis of aMCI met the Petersen criteria, which is based on cognitive impairments that predominantly affect memory in the absence of dementia or significant functional loss [35], and a clinical dementia rating (CDR) score of 0.5 [36]. All subjects satisfied the following criteria: (1) age of 50–90 years; (2) ability to cooperatively finish all tests; (3) free of definite stroke history; and (4) free of any serious medical, neurological (except for AD) or psychiatric disorders, or a history of brain injury. We excluded mixed dementia and other brain disorders based on conventional MR images. Two experienced radiologists assessed conventional MR images of each subject and excluded subjects who satisfied any of the following criteria: (1) with any brain lesions except for lacunar infarction and white matter hyperintensity; (2) with more than one lacunar infarction which is defined as a maximal lesion diameter of <1 cm; and (3) with moderate to severe white matter hyperintensity as assessed by a Fazekas scale of >2. The Fazekas scale is a widely used measure (grades from 0 to 6) to assess the severity of white matter hyperintensity [37]. These older subjects were categorized into 28 NC, 20 aMCI patients, and 39 AD patients. Thirteen additional subjects were excluded due to excessive head motion during MR scanning or poor image quality. In total, 74 subjects, including 21 NC, 18 aMCI, and 35 AD, were included in the further analyses. The demographic and neuropsychological data for the 74 subjects are shown in Table 1.

**Table 1:** Demographic information and clinical measures of the NC, aMCI, and AD groups.

	NC	aMCI	AD	*P* values
Number of subjects	21	18	35	
Gender(males/females)	7/14	10/8	17/18	0.348
Age (years)	65.0±8.1	70.2±7.9	65.8±8.3	<0.001
Years of education	11.0±4.4	9.4±4.8	10.6±4.2	0.030
MMSE	28.5±1.4	21.9±5.0	10.1±6.7	<0.001
CDR	0	0.5	1.63±0.69	

Data are shown as the means ± the standard deviations. The *P* values refer to analysis of variance or chi square tests. Abbreviations: AD, Alzheimer’s disease; aMCI, amnestic mild cognitive impairment; CDR, Clinical Dementia Rating; MMSE, Mini-Mental State Examination; NC, normal control.

### MRI Acquisition

MR images were acquired on a 3.0 Tesla MR scanner (Magnetom Trio, Siemens, Germany). Resting-state fMRI scans were acquired with an echo planar imaging (EPI) sequence with the following scan parameters: repetition time (TR) = 2000 ms, echo time (TE) = 30 ms, flip angle (FA) = 90°, matrix = 64×64, field of view (FOV) = 220×220 mm^2^, slice thickness = 3 mm, and slice gap = 1 mm. Each brain volume comprised 32 axial slices, and 180 volumes were acquired. During fMRI scans, all subjects were instructed to keep their eyes closed, to stay as motionless as possible, to think of nothing in particular, and not to fall asleep. Sagittal T1-weighted MR images were acquired by a magnetization prepared rapid gradient echo (MP-RAGE) sequence (TR/TE = 2000/2.6 ms; FA = 9°; matrix = 256×224; FOV = 256×224 mm^2^; inversion time = 900 ms; slice thickness = 1 mm, no gap; 176 slices; a voxel size of 1 mm×1 mm×1 mm).

### Data Preprocessing

#### fMRI preprocessing

The fMRI data were analyzed using the Data Processing Assistant for Resting-State fMRI (DPARSFA) [38]. The first 10 volumes from each subject were discarded to allow the signal reach equilibrium and the participants adapt to the scanning noise. The remaining 170 volumes were corrected for acquisition time delay between different slices. Then, head motion parameters were estimated, and each volume was realigned to the mean map of the whole volume to correct for geometrical displacements using a six-parameter rigid-body transformation. Five subjects were excluded from further analysis because they had maximum displacements in one or more of the orthogonal directions (x, y, z) of >3 mm or a maximum rotation (x, y, z) >3.0°. The data were spatially normalized to the standard EPI template and re-sampled to 2-mm^3^ voxels. The normalized data were smoothed with a 4 mm full-width at half-maximum (FWHM) Gaussian kernel.

#### Identification of RSNs

We performed ICA using the group ICA (GICA) of the fMRI toolbox (Stable and Consistent Group ICA of the fMRI Toolbox, version 1.2; http://www.nitrc.org/projects/cogicat/) that was established for the analysis of fMRI data. Recently, Zhang et al. found that in multi-stage principal component analysis (PCA) reduction, which is adopted and implemented in GIFT [39] and MELODIC [40], different subject concatenation orders (SCOs) produce variation in the GICA results. To achieve robust and accurate results, an improved algorithm, the Subject Order Independent Group ICA (SOI-GICA) [41], was implemented multiple times with randomized initial values and different subject orders. Then, the multiple results were integrated to form the final output. The toolbox supports a GICA approach that first concatenates the individual data across time and subsequently computes the subject specific components and time courses. The toolbox performed the analysis in three stages: (*i*) data reduction, (*ii*) application of the ICA algorithm, and (*iii*) back-reconstruction for each individual subject. In the present study, we adopted the SOI-GICA, performed GICA 100 times, and obtained 20 independent components (ICs). Eight meaningful components were identified as RSNs via visual inspection. The individual-level components were obtained from back-reconstruction and converted into z-scores, which reflect the degree to which the time series of a given voxel correlates with the mean time series of the component to which it belongs.

#### ALFF calculation

The ALFF was computed using the DPARSFA [38]. Because the ALFF represents the low-frequency band, linear-trend removing and temporal band-pass filtering (0.01–0.08 Hz) were performed on the time series of each voxel to reduce the effects of very-low-frequency drift and high-frequency noise [42], [43]. Then, the time series of each voxel was transformed to the frequency domain using the fast Fourier transform (parameters: taper percent = 0, length = shortest), and the power spectrum was obtained. The square root of the power spectrum was calculated at each frequency and averaged across 0.01–0.08 Hz for each voxel. This averaged square root was taken as the ALFF [44]. For standardization purposes, the ALFF of each voxel was divided by the global mean ALFF within the brain tissue mask. The standardized ALFF of each voxel should have a value of approximately 1, and this standardization procedure is analogous to that used in PET studies [45]. Finally, spatial smoothing was conducted on the standardized ALFF map of each subject with an isotropic Gaussian kernel of 4 mm full-width at half-maximum.

#### Structural MRI preprocessing

Voxel-based morphometry (VBM) analysis was performed using Statistical Parametric Mapping (SPM8; http://www.fil.ion.ucl.ac.uk/spm/software/spm8). The structural MR images were segmented into grey matter (GM), white matter and cerebrospinal fluid [46]. Following segmentation, GM population templates were generated from the entire image dataset using diffeomorphic anatomical registration through the exponentiated Lie algebra (DARTEL) technique [47]. After an initial affine registration of the GM DARTEL template to the tissue probability map in Montreal Neurological Institute (MNI) space (http://www.mni.mcgill.ca/), non-linear warping of GM images was performed to the DARTEL GM template in MNI space with a resolution of 1.5-mm^3^ (as recommended for the DARTEL procedure). The GMV of each voxel was obtained by multiplying the GM concentration map by the non-linear determinants derived from the spatial normalization step. The GMVs represent the probability that each voxel is grey matter with a correction for individual brain sizes. Finally, to compensate for residual between-subject anatomical differences, the GMV images were smoothed with a FWHM kernel of 4 mm. In effect, the analysis of modulated data tests for regional differences in the absolute volume of the brain and removes the confounding effect of variance in individual brain sizes. After spatial pre-processing, the smoothed, modulated, normalized GMV maps were used for statistical analysis.

### Statistical Analysis

**Figure 1:**
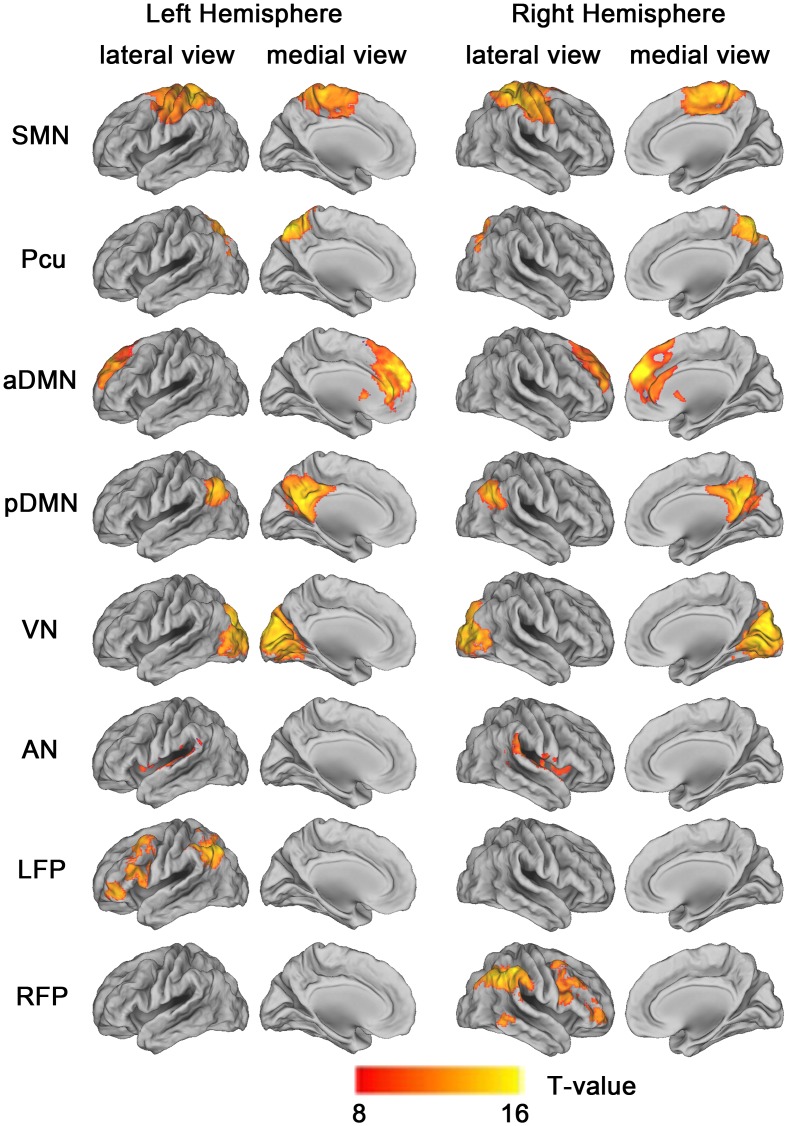
Cortical representation of the 8 resting state networks (RSNs) identified by independent component analysis. Data are displayed on the lateral and medial surfaces of the left and right hemispheres of a brain surface map using CARET software [106]. The color scale represents T values in each RSN. Abbreviations: aDMN, anterior default mode network; AN, auditory network; LFP, left frontoparietal network; Pcu, precuneus network; pDMN, posterior default mode network; RFP, right frontoparietal network; SMN, sensorimotor network; VN, visual network.

All statistical analysis in this study, other than those included in the MRI analysis tools, were performed using Statistical Package for the Social Sciences version 16.0 (SPSS, Chicago, Ill). Post hoc contrasts were tested with the Bonferroni correction (*P*<0.05) for multiple comparisons. ICA components representing RSNs were entered into a one-sample random-effect analyses in SPM8 using a family-wise error (FWE) correction (*P*<0.05 and T = 8) and a cluster size of >100 voxels, to create a sample-specific component map (Fig. 1). All of the following statistical analyses were performed with age, sex, and years of education as covariates of no interest.

The FNC was computed for each pair of the 8 RSNs. We first extracted the mean time series of each RSN of each subject. Then FNC between each pair of the RSNs was calculated using pair-wise correlation between the mean time series of the two RSNs [48]. A one-way analysis of variance (ANOVA) was used to test which pairs of inter-network FC (FNC) showed significant differences across the three groups. Multiple comparisons were corrected using the Bonferroni method (*P*<0.05, corrected). The uncorrected *P*<(corrected *P* = 0.05)/(number of comparisons = 28). When a significant group difference was detected, a post hoc comparison was performed to test the differences in FNCs between every two groups (*P*<0.05, Bonferroni correction; uncorrected *P* value = 0.05/3 = 0.017).

For the intra-network analyses, we first compared the intra-network FCs among the three groups in a voxel-wise manner using ANOVA with a false discovery rate (FDR) corrected threshold of *P*<0.05 and a cluster size of >20 voxels. To further determine whether brain areas with impaired intra-network FC in AD also exhibited alterations in GMV or regional brain activity as assessed by ALFF, brain regions whose intra-network FCs showed significant differences among the three groups were extracted as regions of interest (ROIs). After registration of the FC, ALFF, and GMV maps and ROI resampling, the ALFF and GMV of each ROI were extracted and compared among the three groups using ANOVA and post hoc analysis. To exclude the atrophy effect on the FC and ALFF comparisons, we also repeated the ROI-based analyses of FC and ALFF by controlling for the GMV of each ROI.

Finally, partial correlation analysis was performed to investigate the association between the GMV of each ROI and the MMSE scores in AD and aMCI groups with age, sex, and years of education as covariates of no interest. Similar analyses were also performed for the intra-network FC and ALFF. Moreover, the same analyses for the functional measures were repeated after treating the GMV of each ROI as an additional covariate of no interest. Partial correlation analysis was also performed for the inter-network FC, with and without treating the mean GMV of the whole brain as an additional covariate of no interest. The statistical threshold for these correlation analyses was *P*<0.05.

## Results

### Components of the RSNs

Eight RSNs were identified by the SOI-GICA technique. The RSNs were the SMN, VN, AN, precuneus (Pcu), anterior (aDMN) and posterior (pDMN) DMN, and left (LFP) and right (RFP) FPN (Fig. 1). The components and locations of each RSN were consistent with previous studies [27], [49].

### Aberrant FC between RSNs

**Figure 2:**
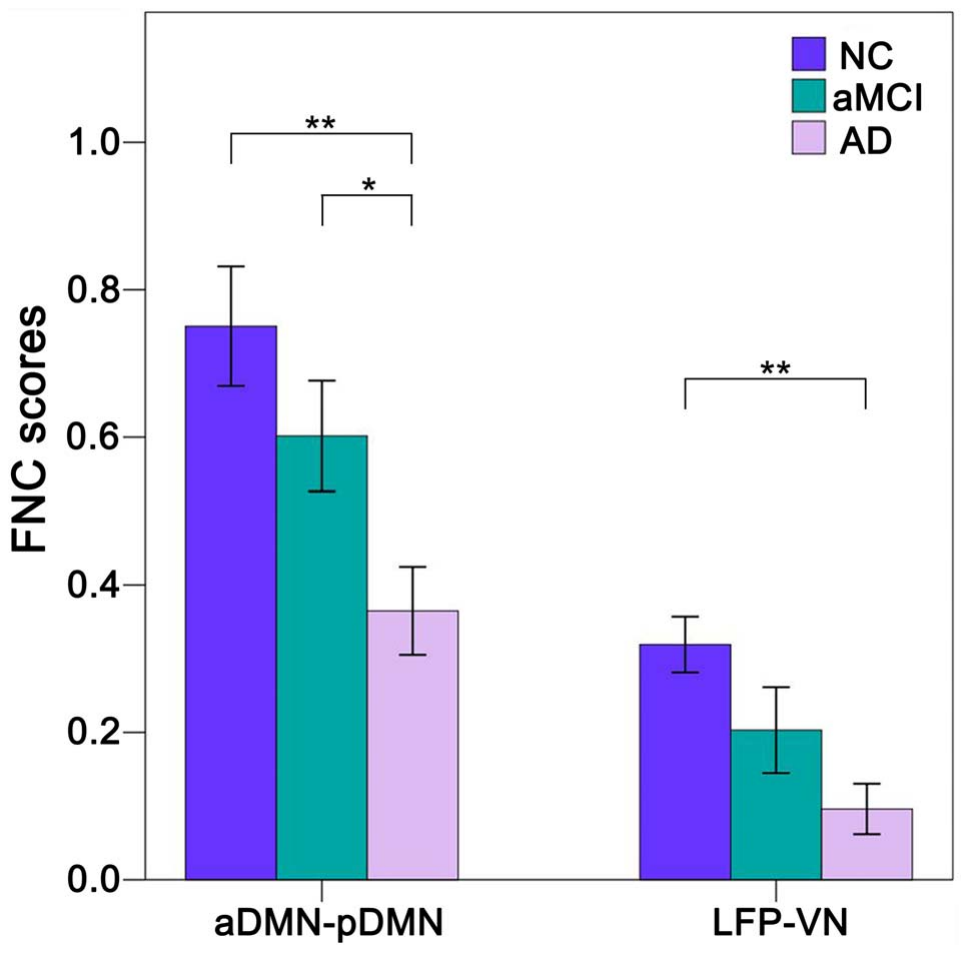
Group differences in functional network connectivity (FNC). The *x*-axis represents the pairs of networks, and the *y*-axis represents the strength of the FNC. Error bars indicate the standard errors of the means. *P<0.05, uncorrected; **P<0.05, Bonferroni corrected. Abbreviations: AD, Alzheimer’s disease; aDMN, anterior default mode network; aMCI, amnestic mild cognitive impairment; LFP, left frontoparietal network; NC, normal controls; pDMN, posterior default mode network; VN, visual network.

Compared with the NC group, AD patients showed significantly (*P*<0.05, Bonferroni correction) decreased FNCs between the aDMN and pDMN and between the VN and LFP. The values of the aMCI group were numerically between the NC and AD groups (Fig. 2). Although there were no significant (*P*<0.05, Bonferroni correction) differences in the FNCs of the NC and aMCI patients or the MCI and AD patients, AD patients showed a trend toward decreased (*P*<0.05, uncorrected) FNC between the aDMN and pDMN compared to the aMCI patients.

### Aberrant FC within RSNs

**Figure 3:**
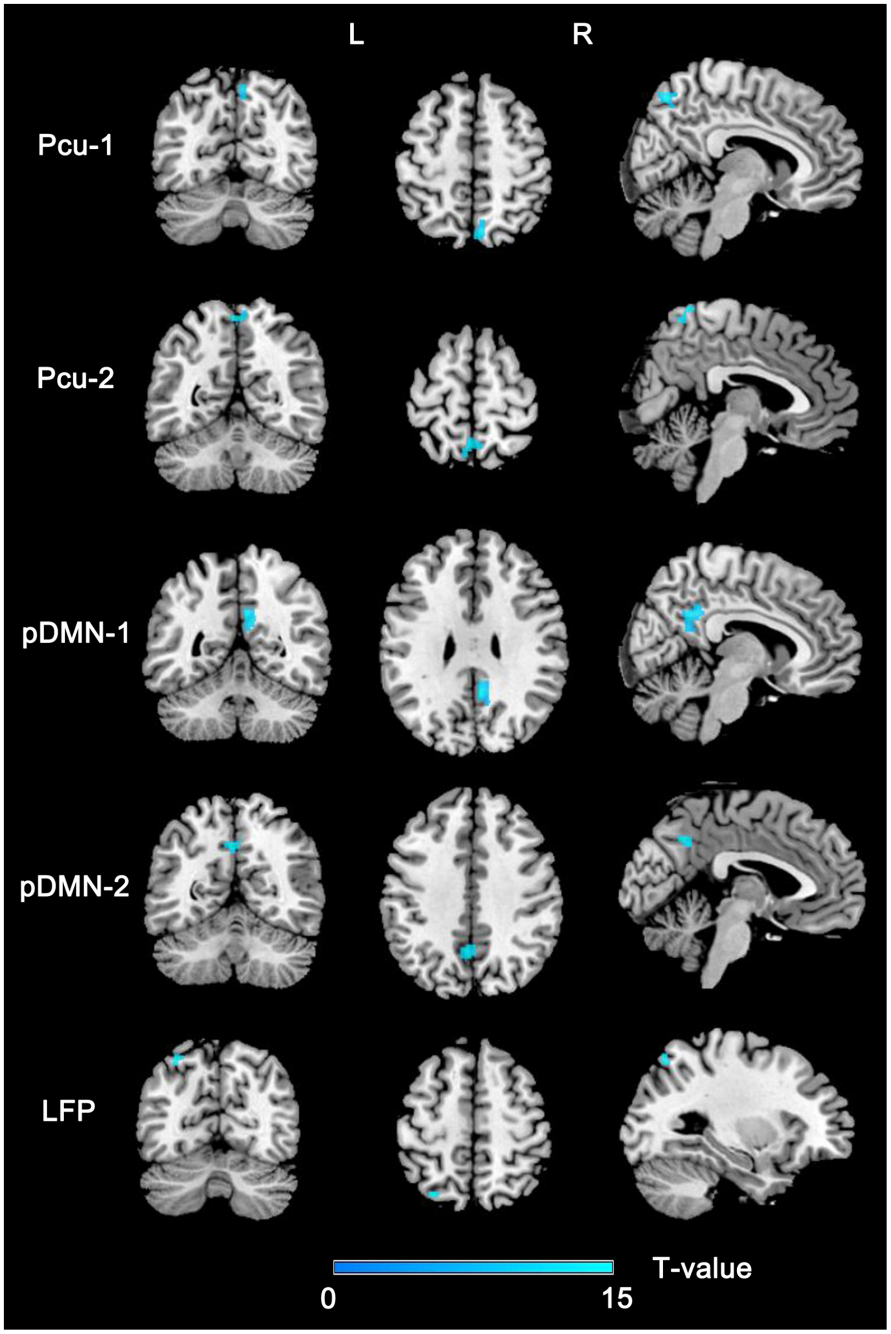
Brain regions with significant differences in the intra-network FC across groups. Abbreviations: FC, functional connectivity; L, left; LFP, left frontoparietal network; Pcu, precuneus network; pDMN, posterior default mode network; R, right.

**Figure 4:**
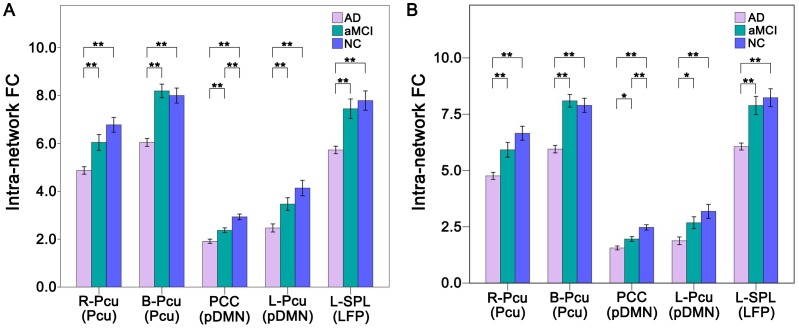
FC differences within the RSNs across groups without (A) and with (B) GMV correction. The *x*-axis represents the brain regions of the RSNs (parenthesis), and the *y*-axis represents the strength of intra-network FC. Error bars indicate the standard errors of the means. *P<0.05, uncorrected; **P<0.05, Bonferroni correction. Abbreviations: AD, Alzheimer’s disease; aMCI, amnestic mild cognitive impairment; B, bilateral; FC, Functional connectivity; GMV, grey matter volume; L, left; LFP, left frontoparietal network; NC, normal controls; Pcu, precuneus; pDMN, posterior default mode network; PCC, posterior cingulate cortex; R, right; SPL, superior parietal lobule.

We compared the intra-network FCs among the three groups in a voxel-wise manner and extracted these significant brain regions as ROIs (Fig. 3 and Table 2). AD patients showed significantly decreased FCs in the bilateral (B-Pcu) and right (R-Pcu) Pcu of the Pcu network, the posterior cingulate cortex (PCC) and left Pcu (L-Pcu) of the pDMN, and the left superior parietal lobule (L-SPL) of the left FPN compared with the NCs (Fig. 4A). All of the comparisons between aMCI and AD patients were significant (*P*<0.05, Bonferroni correction). The FC values of these ROIs of aMCI patients were numerically between those of the NCs and AD patients. Compared with NCs, aMCI patients showed significantly (*P*<0.05, Bonferroni correction) decreased FC in the PCC.

**Table 2:** Brain areas with significant differences in FC across groups.

RSN	Regions	BA	Cluster size (voxels)	Peak *z*-score	MNI Coordination (x, y, z)
Pcu	R-Pcu	7	67	14.26	6, −70, 54
Pcu	B-Pcu	7	86	12.73	0, −54, 62
pDMN	PCC	23	155	15.76	6, −52, 30
pDMN	L-Pcu	7	64	11.44	−2, −58, 40
LFP	L-SPL	7	22	13.39	−28, −68, 56

Abbreviations: AD, Alzheimer’s disease; B, bilateral; BA, Brodmann’s area; FC, functional connectivity; L, left; LFP, left frontoparietal network; MNI, Montreal Neurological Institute; NC, normal controls; PCC, posterior cingulate cortex; Pcu, precuneus; pDMN, posterior default mode network; R, right; RSN, resting-state network; SPL, superior parietal lobule.

AD patients had significant decreases in GMV in several ROIs, such as the PCC and Pcu, which could explain the intra-network FC differences between groups [50]. Thus, we repeated the ROI-based intra-network FC comparisons while controlling for the GMV of each ROI. After GMV correction, compared with aMCI patients, AD patients showed only a trend toward decreased (*P*<0.05, uncorrected) FCs in the ROIs of the pDMN, and the other results did not change (Fig. 4B).

### Aberrant ALFF within RSNs

**Figure 5:**
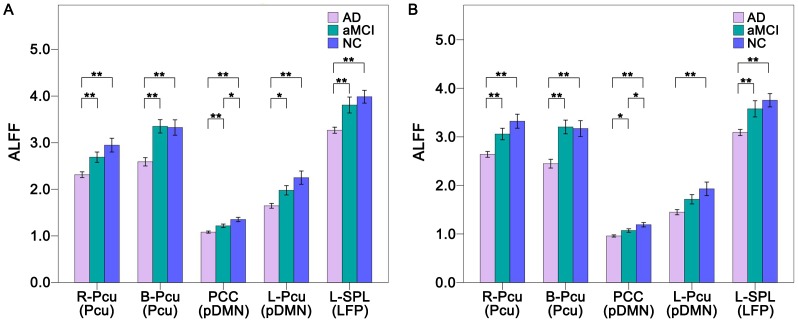
ALFF differences within the RSNs across groups without (A) and with (B) GMV correction. The *x*-axis represents the brain regions of the RSNs (parenthesis), and the *y*-axis represents the ALFF. Error bars indicate the standard errors of the means. *P<0.05, uncorrected; **P<0.05, Bonferroni corrected. Abbreviations: AD, Alzheimer’s disease; ALFF, amplitude of low-frequency fluctuation; aMCI, amnestic mild cognitive impairment; B, bilateral; GMV, grey matter volume; L, left; LFP, left frontoparietal network; NC, normal controls; Pcu, precuneus; pDMN, posterior default mode network; PCC, posterior cingulate cortex; R, right; SPL, superior parietal lobule.

We also performed ROI-based ALFF analysis to reveal differences in regional brain activity across the three groups. Compared with the NC, we found that the ALFF was significantly (*P*<0.05, Bonferroni correction) decreased in all five ROIs of the AD patients. Compared with aMCI patients, AD patients showed significantly (*P*<0.05, Bonferroni correction) decreased ALFF in the R-Pcu, B-Pcu, PCC and L-SPL and exhibited a trend toward decreased (*P*<0.05, uncorrected) ALFF in the L-Pcu. The aMCI group showed a trend toward decreased (*P*<0.05, uncorrected) ALFF in the PCC relative to the NC group. Notably, the ALFFs of the aMCI group were numerically between the NC and AD groups (Fig. 5A). After GMV correction, most of the between-group differences in ALFF remained (*P*<0.05, Bonferroni correction) except for the ROIs of the pDMN between the aMCI and AD groups. That is, the AD group showed a trend toward decreases (*P*<0.05, uncorrected) ALFF in the PCC compared to the aMCI group, and no significant difference (*P*<0.05, uncorrected) in ALFF between the aMCI and AD groups was found in the L-Pcu (Fig. 5B).

### Aberrant GMV within RSNs

**Figure 6:**
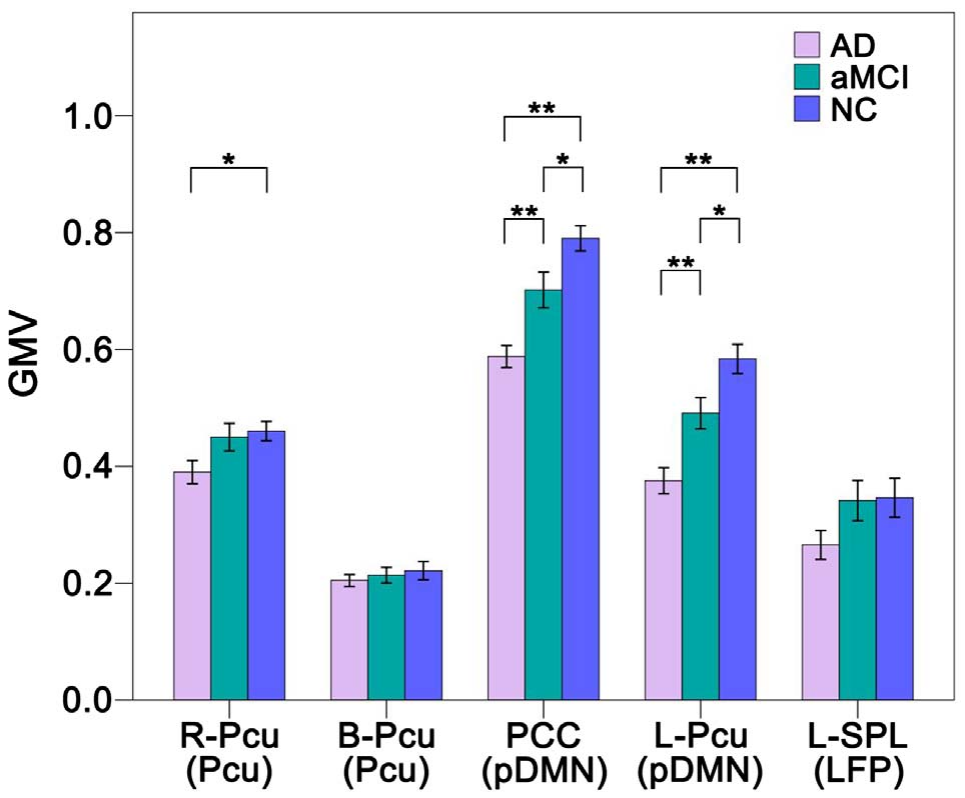
Grey matter volume (GMV) differences within the RSNs across groups. The *x*-axis represents the brain regions of the RSNs (parenthesis), and the *y*-axis represents the GMV. Error bars indicate the standard errors of the means. *P<0.05, uncorrected; **P<0.05, Bonferroni corrected. Abbreviations: AD, Alzheimer’s disease; aMCI, amnestic mild cognitive impairment; B, bilateral; L, left; LFP, left frontoparietal network; NC, normal controls; Pcu, precuneus; pDMN, posterior default mode network; PCC, posterior cingulate cortex; R, right; SPL, superior parietal lobule.

The GMV comparisons across the three groups are shown in Fig. 6. Compared with NCs, patients with AD showed significant (*P*<0.05, Bonferroni correction) GMV decreases in the PCC and L-Pcu, and a trend toward decreased (*P*<0.05, uncorrected) GMV in the R-Pcu. Compared with aMCI patients, AD patients showed significantly decreased GMV in the PCC and L-Pcu. The aMCI group showed a trend toward decreased (*P*<0.05, uncorrected) GMV in the PCC and L-Pcu compared with the NC group. The B-Pcu and L-SPL did not show any significant (*P*<0.05, uncorrected) changes in GMV across the three groups.

### Differences in ALFF and GMV across the Whole Brain

Considering that the ROI-based analyses of the ALFF and GMV cannot provide a full picture of the whole brain changes, we also compared the ALFF and GMV among AD, aMCI and NC groups in a voxel-wise manner of the whole brain using ANOVA. Significant ALFF differences (*P*<0.005, uncorrected) among the three groups are found in the multiple brain regions (Table S1 and Figure S1). To test whether the ROIs with significant differences in the intra-network FCs also show significant differences in ALFF, we overlaid these ROIs onto the maps that show significant differences in the ALFF (Figure S2). We found that most of these ROIs were overlapped with brain regions with significant differences in the ALFF (Figure S2). In the voxel-based GMV analysis (*P*<0.05, FDR corrected), we found widespread brain regions showed significant GMV differences among the three groups (Figure S3), including several ROIs with significant differences in intra-network FC, such as the PCC/Pcu.

### Correlations between MRI Indices and MMSE

**Figure 7:**
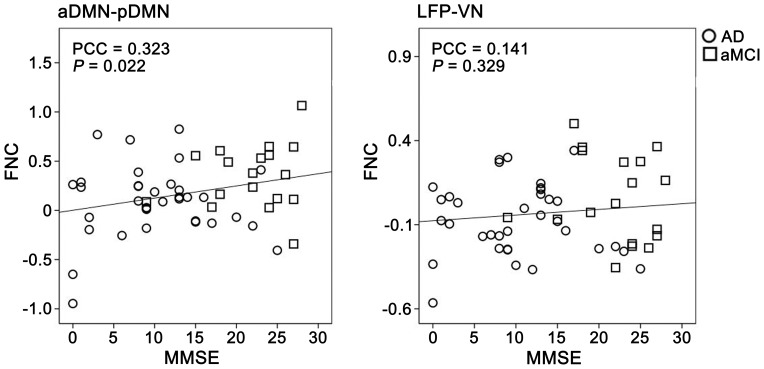
Scatter plots of FNCs versus MMSE scores. Abbreviations: AD, Alzheimer’s disease; aDMN, anterior default-mode network; aMCI, amnestic mild cognitive impairment; FNC, functional network connectivity; LFP, left frontoparietal network; MMSE, Mini-Mental State Examination; pDMN, posterior default mode network; PCC, partial correlation coefficient; VN, visual network.

**Figure 8:**
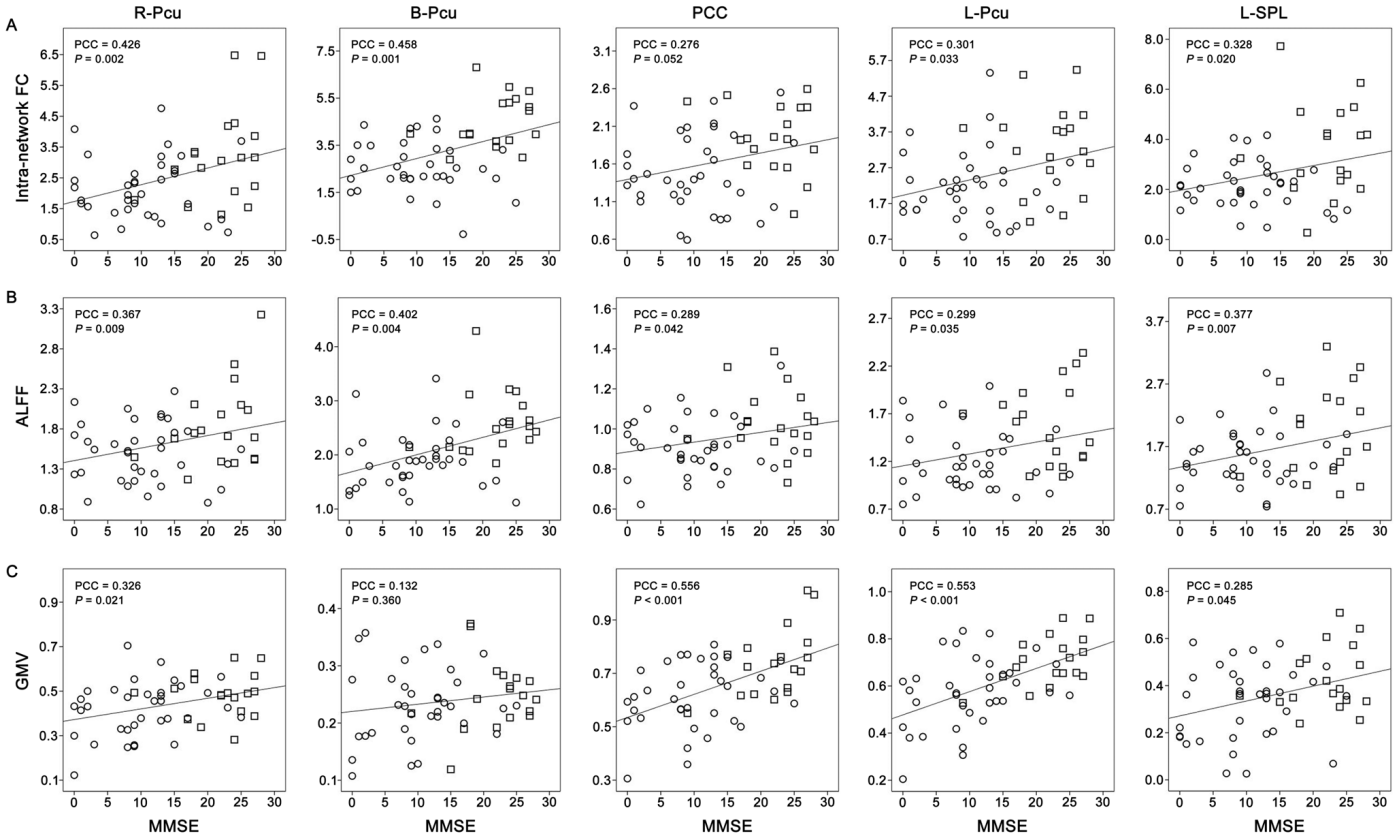
Scatter plots of intra-network FCs (A), ALFFs (B), and GMVs (C) versus MMSE scores. The circles represent the Alzheimer’s disease subjects and the squares represent the amnestic mild cognitive impairment subjects. Abbreviations: ALFF, amplitude of low-frequency fluctuation; B, bilateral; FC, functional connectivity; GMV, grey matter volume; L, left; MMSE, Mini-Mental State Examination; PCC, partial correlation coefficient; PCC^a^, posterior cingulate cortex; Pcu, precuneus; R, right; SPL, superior parietal lobule.

To determine whether MRI indices with significant group differences contributed to the decline in cognitive function in patients, we performed partial correlation analyses between these MRI indices and MMSE scores. We found that most of the FCs, ALFFs, and GMVs of the ROIs were correlated with MMSE scores (*P*<0.05), except for the FNC between the LFP and VN (*P* = 0.329), the FCs of the PCC (*P* = 0.052), the GMV of the B-Pcu (*P* = 0.360) (Fig. 7, Fig. 8, Table 3, and Table 4). After correction for GMV, most of the correlations of functional measures were significant (*P*<0.05), except for the FNC between the aDMN and pDMN (*P* = 0.955), the FCs of the L-Pcu (*P* = 0.142), the ALFF of the PCC (*P* = 0.233) and L-Pcu (*P* = 0.189) (Table 3 and Table 4).

**Table 3:** Correlations between MMSEs and FNCs.

FNC	Without GMV correction		With GMV correction	
	PCC	*P*	PCC	*P*
aDMN-pDMN	0.323	**0.022**	−0.008	0.955
LFP-VN	0.141	0.329	0.277	0.054

Note: *P* values were adjusted for multiple comparisons using the Bonferroni correction.

Abbreviations: aDMN, anterior default mode network; FNC, functional network connectivity; GMV, grey matter volume; LFP, left frontoparietal network; MMSE, Mini-Mental State Examination; PCC, partial correlation coefficient; pDMN, posterior default mode network; VN, visual network.

**Table 4:** Correlations between MMSE scores and functional/structural indices.

Regions	FC				ALFF				GMV	
	Without GMV correction		With GMV correction		Without GMV correction		With GMV correction			
	PCC	*P*	PCC	*P*	PCC	*P*	PCC	*P*	PCC	*P*
R-Pcu	0.426	**0.002**	0.384	**0.006**	0.367	**0.009**	0.370	**0.009**	0.326	**0.021**
B-Pcu	0.458	**0.001**	0.454	**0.001**	0.402	**0.004**	0.403	**0.004**	0.132	0.360
PCC^a^	0.276	0.052	0.163	0.263	0.289	**0.042**	0.174	0.233	0.556	**<0.001**
L-Pcu	0.301	**0.033**	0.213	0.142	0.299	**0.035**	0.191	0.189	0.553	**<0.001**
L-SPL	0.328	**0.020**	0.337	**0.018**	0.377	**0.007**	0.321	**0.025**	0.285	**0.045**

Note: *P* values were adjusted for multiple comparisons using the Bonferroni correction.

Abbreviations: ALFF, amplitude of low-frequency fluctuation; B, bilateral; FC, functional connectivity; GMV, grey matter volume; L, left; MMSE, Mini-Mental State Examination; PCC, partial correlation coefficient; PCC^a^, posterior cingulate cortex; Pcu, precuneus; R, right; SPL, superior parietal lobule.

## Discussion

In the present study, we answered the four questions proposed in the introduction. We found the following: (1) the Pcu, pDMN and LFP networks were selectively impaired in AD, and only the pDMN was impaired in MCI; (2) brain regions with impaired FC in AD also exhibited alterations in ALFF and GMV; (3) most of the functional changes were independent of the structural changes; (4) FNCs between the aDMN and pDMN as well as between the VN and LFP were also changed in AD; and (5) most of these MRI measures were associated with cognitive decline in older people. The results suggest that the brain networks supporting complex cognitive processes are specifically and progressively impaired over the course of AD.

### Multiple RSN Impairments in AD

The main function of the DMN is episodic memory processing [51], which is impaired in AD [4]. The structural and functional impairments within the DMN, as found here, have been frequently reported in AD [19], [25]. The novelty of our study is the finding that the functional deficits of the DMN regions are independent of their structural impairments in AD patients. It is generally assumed that the functional connectivity within the intrinsic networks reflects the direct or indirect structural connectivity. Nonetheless, it’s not a simple one-to-one mapping. Functional connectivity can exist in the absence of structural connectivity. Functional connectivity abnormalities are found at an early stage of some diseases, even before the detectable structural impairments [52]. We found that most of the functional differences remained significant after controlling for GMV, suggesting that the functional deficits in AD cannot be completely explained by the decreased GMV. That is to say, AD is associated with relatively more serious functional damage, which is not only secondary to the structure impairments. These findings suggest that both structural and functional deficits in the PCC and L-Pcu of the DMN contribute to AD pathology.

Beyond the DMN, several other RSNs have been shown to be impaired in AD [22], [25], [27]. In the present study, AD patients showed decreased FC, ALFF, and GMV within the Pcu and the left FPN compared with the NCs. The Pcu network has been identified as an independent RSN in a previous ICA study [49]. This network responds to a wide range of cognitive processes, such as reflective, self-related processing [53], [54], awareness and conscious information processing [55], [56], episodic memory [57]–[59], and visuospatial processing [60], [61]. Our finding of structural and functional impairments in the Pcu network in AD is consistent with the established concept that the precuneus is particularly vulnerable in AD [62]–[68].

As a lateralized RSN, the FPN has been identified by most previous ICA studies of resting-state fMRI data [69]–[71]. The FPN is activated during a wide array of goal-directed cognitive tasks [72]–[74] and is associated with memory [14], language [75], attention [73], [76], and visual [77] processes. As a core node of the FPN, the SPL plays an important role in visual attention [78]–[81]. The decreased FC, ALFF, and GMV in the SPL may underlie deficits in visual attention in AD [82]. Moreover, structural and functional abnormalities in the SPL in AD have been demonstrated in several previous studies and include decreases in GMV and cortical thickness [23], [83], [84], activation [82], resting-state FC [22], and effective connectivity [23]. In direct support of our finding, decreased LFP connectivity has been described in AD [25]. We did not find connectivity changes in the RFP, suggesting the laterality (left) of impaired FPN in AD. The left lateralized FPN impairment in AD is supported by a previous study of resting-state FC changes in the attention network in AD [85]. However, the laterality pattern needs to be further validated.

### Inter-network Connectivity Impairments in AD

As mentioned in the introduction, little is known about the FNC alterations in AD. A recent ROI-based FC study revealed decreased anticorrelations between three pairs of anticorrelated RSNs, the DMN-dorsal attention network, the DMN-SMN, and the control network-SMN [29]. Moreover, a resting-state fMRI study detected three decreased connections (the SMN→self-referential network (SRN), the SRN→ventral attention network (VAN), and the VAN→dorsal attention network (DAN) and one increased connection (DMN→DAN) in AD [30]. However, we used the data-driven method of ICA to identify RSNs and computed FNCs between different pairs of RSNs [30], [31], [86]. We found decreased positive correlations between the aDMN and pDMN and between the LFP and VN. The interaction of the anterior and posterior regions of the DMN may serve to organize neuronal activity [87] or to support mind wandering during self-referential mental processing [88], [89]. Decreases in resting-state FCs between the anterior and posterior portions of the DMN have been well established in AD [90]–[95]. It has been suggested that decreased connectivity between the anterior and posterior components of the DMN may underlie deficits in self-referential processing, attention control and working memory [96].

It has been suggested that the FPN and VN cooperate to support visual attention [97]. A pioneering study revealed that visual cortical areas that selectively process relevant information are functionally connected with the FPN [98], which is associated with top-down enhancement of task-relevant stimuli [72]. The impairment of selective attention, especially visual attention, has been documented in patients with AD [82], [99]–[104]. The decreased FNC between the LFP and VN may be a possible mechanism of impaired visual attention in AD.

### Network Markers of aMCI

Although the values of MRI measures of the aMCI patients were numerically between those of NCs and AD patients, only the PCC of the posterior DMN showed decreased FC, ALFF and GMV in aMCI patients compared with NCs. These findings suggest that the impairment of the PCC occurs as early as the MCI stage, which is consistent with the observations of previous studies [15], [16], [18], [19]. It seems that the PCC is preferentially affected in aMCI patients and could be used as a biological marker to distinguish MCI patients from NCs.

### Biomarkers for Cognitive Decline in Older People

In older people, most of the FCs, ALFFs, and GMVs of the ROIs were correlated with MMSE scores except for the FCs of the PCC and GMV of the B-Pcu. Most of these correlations of functional measures remained significant after GMV correction. These findings suggest that both structural and functional impairments in the cognitive-related RSNs independently contribute to cognitive decline in older people.

### Limitations

It should be noted that the significance of our results are not as high as some previous reports [105], the differences in sample size, demographics, analyzing methods may partly account for the differences in significance across studies. More importantly, our current study is limited by the relatively small number of available samples, especially in the aMCI group, and the large difference in age between the 3 groups. Further studies with a larger sample size and more matched groups should be done to validate our findings.

## Supporting Information

Figure S1 (DOC)

Figure S2 (DOC)

Figure S3 (DOC)

Table S1 (DOC)
