# Supplementary material for: Aberrant Functional Organization within and between Resting-State Networks in AD
Source: PLoS One. 2013 May 7;8(5):e63727. doi: 10.1371/journal.pone.0063727 (PMC3647055; doi:10.1371/journal.pone.0063727)
Supplement: Figure S2 — (DOC) [file pone.0063727.s002.doc]

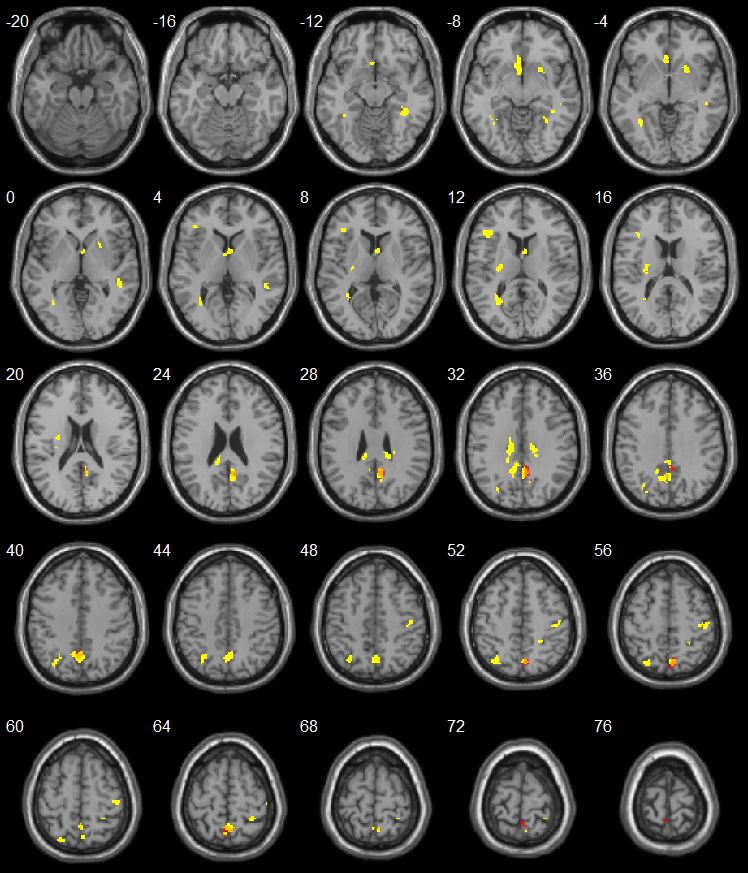


**Figure S2.** Overlaps between ROIs with significant differences in the intra-network FC (red color) and brain regions with significant differences in the ALFF (yellow color). Abbreviations: ALFF, amplitude of low-frequency fluctuation; FC, functional connectivity; ROI, region of interest.
