# Supplementary material for: Aberrant Functional Organization within and between Resting-State Networks in AD
Source: PLoS One. 2013 May 7;8(5):e63727. doi: 10.1371/journal.pone.0063727 (PMC3647055; doi:10.1371/journal.pone.0063727)
Supplement: Figure S3 — (DOC) [file pone.0063727.s003.doc]

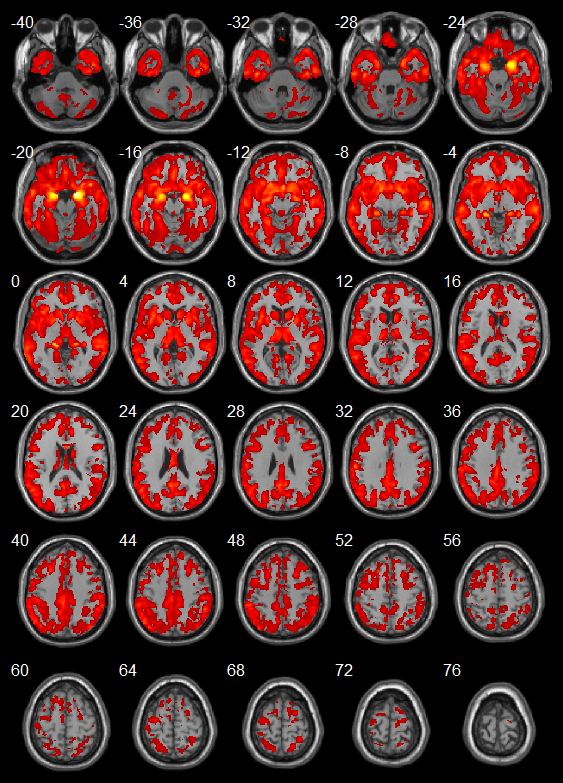


**Figure S3.** Voxel-based analysis of the GMV in the whole brain among the AD, aMCI, and NC groups using one-way ANOVA (*P* < 0.05, FDR corrected). Abbreviations: AD, Alzheimer’s disease; aMCI, amnestic mild cognitive impairment; ANOVA, analysis of variance; FDR, false discovery rate; GMV, gray matter volume; NC, normal controls; ROI, region of interest.
