# Supplementary material for: Aberrant Functional Organization within and between Resting-State Networks in AD
Source: PLoS One. 2013 May 7;8(5):e63727. doi: 10.1371/journal.pone.0063727 (PMC3647055; doi:10.1371/journal.pone.0063727)
Supplement: Table S1 — (DOC) [file pone.0063727.s004.doc]

**Table S1.** Brain areas with differences in ALFF among the AD, aMCI and NC groups (*P* < 0.005, uncorrected).

| Regions | BA | Cluster size (voxels) | Peak *z*-score | MNI Coordination (x, y, z) |
| --- | --- | --- | --- | --- |
| L-inferior frontal gyrus | 45/48 | 84 | 11.93 | -38, 30, 10 |
| ACC | 25/11 | 112 | 14.20 | -4, 24, -4 |
| R-Putamen | 48 | 63 | 14.65 | 24, 10, -4 |
| L-Putamen | 48 | 73 | 10.33 | -26, -16, 14 |
| L-PCC | 23 | 113 | 10.06 | -14, -24, 32 |
| R-PCC | 23 | 75 | 12.24 | 16, -30, 28 |
| R-postcentral gyrus | 3/4 | 95 | 10.39 | 46, -18, 50 |
| R-postcentral gyrus | 2 | 50 | 10.28 | 32, -44, 64 |
| B-PCC | 7/23 | 569 | 16.29 | 8, -52, 24 |
| B-precuneus | 7 | 94 | 9.54 | -2, -54, 66 |
| L-SPL | 7/19 | 209 | 18.26 | -30, -64, 48 |
| R-fusiform gyrus | 37 | 67 | 10.27 | 38, -40, -10 |
| L-fusiform gyrus | 37 | 138 | 14.24 | -30, -54, 12 |
| R-MTG | 21 | 65 | 10.99 | 48, -32, -2 |

Abbreviations: ACC, Anterior cingulate cortex; AD, Alzheimer’s disease; ALFF, amplitude of low-frequency fluctuation; aMCI, amnestic mild cognitive impairment; B, bilateral; BA, Brodmann’s area; L, left; MNI, Montreal Neurological Institute; MTG, Middle temporal gyrus; NC, normal controls; PCC, posterior cingulate cortex; R, right; SPL, superior parietal lobule.
